# Clinicopathological and Genetic Profiles of Cases with Myocyte Disarray—Investigation for Establishing the Autopsy Diagnostic Criteria for Hypertrophic Cardiomyopathy

**DOI:** 10.3390/jcm8040463

**Published:** 2019-04-05

**Authors:** Yukiko Hata, Shojiro Ichimata, Yoshiaki Yamaguchi, Keiichi Hirono, Yuko Oku, Fukiko Ichida, Naoki Nishida

**Affiliations:** 1 Department of Legal Medicine, Graduate School of Medicine and Pharmaceutical Sciences, University of Toyama, Toyama 930-0194, Japan; yhatalm@med.u-toyama.ac.jp (Y.H.); ichimata@med.u-toyama.ac.jp (S.I.); yoku@med.u-toyama.ac.jp (Y.O.); 2 Second Department of Internal Medicine, Graduate School of Medicine and Pharmaceutical Sciences, University of Toyama, Toyama 930-0194, Japan; y.yoshiaki.i0721@gmail.com; 3 Department of Pediatrics, Graduate School of Medicine and Pharmaceutical Sciences, University of Toyama, Toyama 930-0194, Japan; khirono1973@gmail.com (K.H.); fkichida@iuhw.ac.jp (F.I.)

**Keywords:** autopsy, diagnostic criteria, hypertrophic cardiomyopathy, myocytes disarray, next-generation sequencing

## Abstract

Myocyte disarray of >10% in the heart is broadly accepted as a diagnostic pitfall for hypertrophic cardiomyopathy (HCM) at postmortem. The present study aims to propose an additional diagnostic criterion of HCM. Heart specimens from 1387 serial forensic autopsy cases were examined. Cases with myocyte disarray were extracted and applied to morphometric analysis to determine the amount of myocyte disarray. Comprehensive genetic analysis by using next-generation sequencing was subsequently applied for cases with myocyte disarray. Fifteen cases with myocyte disarray were extracted as candidate cases (1.1%, 11 men and 4 women, aged 48–94 years). In terms of the cause of death, only 2 cases were cardiac or possible cardiac death, and the other was non-cardiac death. Six cases showed myocyte disarray of >10% and 3 cases showed myocyte disarray of 5% to 10%. The other 6 cases showed myocyte disarray of <5%. Nine rare variants in 5 HCM-related genes (*MYBPC3, MYH7, MYH6, PRKAG2,* and *CAV3*) were found in 8 of 9 cases with myocyte disarray of >5%. The remaining 1 and 6 cases with myocyte disarray of <5% did not have any such variant. Myocyte disarray of >5% with rare variants in related genes might be an appropriate postmortem diagnostic criterion for HCM, in addition to myocyte disarray of 10%.

## 1. Introduction

Hypertrophic cardiomyopathy (HCM) is a common autosomal dominant genetic disorder, and is characterized macroscopically through left ventricular hypertrophy [1]. The histological hallmark of HCM is the triad of myocyte hypertrophy, myocyte disarray, and interstitial fibrosis. Myocyte disarray is characterized by architectural disorganization of the myocardium in which adjacent hypertrophied myocytes are aligned perpendicularity or obliquely to each other around the central core of collagen in a pinwheel configuration. This is the most reliable finding for pathological diagnosis of HCM [2]. However, while myocyte disarray of >10% in the heart has been accepted as a point of consensus for HCM, [2,3] the diagnostic significance of a lower percentage of myocyte disarray has not been determined yet.

The left ventricular hypertrophy in HCM may be symmetrical or asymmetrical, [4] and HCM may affect any portion of the left ventricle. Such heterogeneity may lead to a wide variation in morphological and clinical manifestations of HCM [4,5]. The typical classical anatomical form was initially shown by Teare in 1958, who found that the heart showed narrowing of the left ventricular outflow tract due to thickening of the basal anterior septum [6]. Furthermore, several studies have shown unusual and rare morphological variants, including cases with a mid-ventricular obstructive form, [7] apical form, [8] or even cases without left ventricular hypertrophy [9]. Therefore, a histopathological examination should be essential for a definite diagnosis of HCM [2]. Previous autopsy studies have shown that there are confusing cases with asymmetric exaggeration of the basal anterior septum due to hypertension and aortic valve stenosis, and a physiological sigmoid septum occurs when the take-off of the ascending aorta from the left ventricle is sharply angled [2,10,11].

The recent development of next-generation sequencing (NGS) has facilitated genetic testing of multiple inherited cardiac disorder-associated genes, including channelopathy-, cardiomyopathy-, or other heart disease-associated genes [12]. Some studies, including our study, showed that genetic investigation using NGS had substantial potential for a correct postmortem diagnosis, regardless of the macroscopic appearance of the heart or the clinical history of autopsied cases [13,14]. HCM is frequently described as a disease of the sarcomere, with pathogenic variants detected in almost all sarcomeric proteins [15].

The prevalence of HCM examined by echocardiographic analysis is 1:500 to 1:200 in the general population of healthy young adults [16]. We previously showed that a large cohort of forensic autopsy cases, without case selection, may be useful for examining the prevalence, and early clinical and pathological manifestations of neurodegenerative disease [17,18]. In the current study, we examined serial forensic autopsy cases, and performed a detailed pathological examination to detect cases with myocardial disarray, and subsequently performed genetic analysis for detected cases. This study aimed to investigate the prevalence of undiagnosed HCM in the Japanese population after establishing postmortem diagnostic criteria of HCM. We also examined the pathological and genetic features of such cases to discuss the phenotype–genotype correlation in undiagnosed HCM cases.

## 2. Methods

All autopsies performed in our department from January of 2010 to September of 2018 were evaluated (*n* = 1506). Of these, heart specimens from 1387 autopsies (0–101 years old; males, 871; females, 516; mean age, 63.1 ± 21.1 years) without severe injury or postmortem degradation were examined. A natural cause of death was recorded for 339 cases (males, 245; females, 94), and 579 cases (males, 389; females, 190) suffered from accidental traumatic death (e.g., fall, traffic accident, burning, drowning, or hypothermia). Suicide or homicide accounted for 426 cases (males, 214; females, 212), and there were 43 cases (males, 24; females, 19) with undetermined causes of death. The clinical history of patients was obtained from their families and from police records. Toxicological screening was applied to all cases and quantitative assessment was also performed as appropriate. The ethical committee of Toyama University approved this study, which was performed in accordance with the ethical standards established in the 1964 Declaration of Helsinki.

The hearts were excised and dissected free from the great vessels. Heart weight, including epicardial coronary arteries and epicardial fat, was measured to the nearest gram. The right and left ventricles were cut at 1 cm intervals parallel to the levels of the papillary muscle from the apex. Sections at the level of the papillary muscle and the level just above the apex were subjected to a thorough histological examination. The major epicardial coronary arteries and the left main, left anterior descending, left circumflex, and right coronary arteries were cut transversely at 5 mm intervals. Hematoxylin–eosin and Masson–Trichrome staining were applied for all specimens [13,14].

Cases that fulfilled the criteria of type Ia myocardial disarray/disorganization as defined by Maron et al. were extracted [19]. The ventricle was divided into 5 areas, which were anterior, lateral, and posterior of the left ventricle, the ventricular septum, and the right ventricle. The presence or absence of myocyte disarray in each case was recorded. The severity of interstitial fibrosis was scored by an investigation of specimens that were stained with the Masson–Trichrome method. No interstitial fibrosis was categorized as grade 0, mild interstitial fibrosis was grade 1, moderate interstitial fibrosis was grade 2, and advanced interstitial fibrosis or replacement fibrosis was grade 3 (Supplemental Figure S1). The relative amount of disarray in whole specimens at the level of the papillary muscle was measured using a BX51 microscope (Olympus, Tokyo, Japan), which was equipped with a digital camera (DP73; Olympus, Tokyo, Japan), and analyzed using Olympus cellSens imaging software (version 1.7, Olympus, Tokyo, Japan). In the present study, disarrangement of myofibers at the junction of the ventricular septum was considered as a physiological appearance [20]. Therefore, this finding was not evaluated as myocyte disarray.

### 2.1. Genetic Testing Using NGS

Genomic DNA was extracted from whole blood samples from autopsy cases with myocardial disarray using a QIAamp DNA Mini Kit (Qiagen, Redwood City, CA, USA). We designed a custom AmpliSeq panel of PCR primers (Life Technologies, Carlsbad, CA, USA) using Ion AmpliSeq Designer software (Life Technologies, Carlsbad, CA, USA) to target all exons of 81 cardiovascular disorder-related genes (Life Technologies, Carlsbad, CA, USA), which are listed in Supplemental Table S1 was performed using an Ion PGM system (Life Technologies, Carlsbad, CA, USA). This custom panel, which consisted of two separate PCR primer pools and produced a total of 2890 amplicons, was used to generate target amplicon libraries. Genomic DNA samples were each PCR-amplified using the custom-designed panel and Ion AmpliSeq Library Kit Plus (Life Technologies, Carlsbad, CA, USA) in accordance with the manufacturer’s instructions. Various samples were distinguished using an Ion Xpress Barcode Adapters Kit (Life Technologies, Carlsbad, CA, USA) and then pooled in equimolar concentrations. Emulsion PCR and Ion Sphere Particle (ISP) enrichment were performed with an Ion PGM Hi-Q View OT2 200 Kit (Life Technologies, Carlsbad, CA, USA) in accordance with the manufacturer’s instructions. ISPs were loaded on an Ion 318 Chip v2 and sequenced with an Ion PGM Hi-Q View Sequencing Kit (Life Technologies, Carlsbad, CA, USA). Minimal coverage of 20 reads was defined as the cut-off value.

All variants derived from PGM sequencing were prioritized and then confirmed by Sanger sequencing to validate the NGS results. For Sanger sequencing, the nucleotide sequences of the amplified fragments were analyzed by direct sequencing in both directions using a BigDye Terminator v3.1 Cycle Sequencing Kit (Applied Biosystems, Foster City, CA, USA) and an ABI 3130xl automated sequencer (Applied Biosystems, Foster City, CA, USA).

### 2.2. Variant Classification of Inherited Genetic Defects

The allelic frequency in the Genome Aggregation Database (gnomAD) was determined for all detected variants and those with a minor allelic frequency of >0.1% in the general population were removed [21]. Variants were then verified using Combined Annotation Dependent Depletion (CADD), which is a tool for scoring variants, and variants with a score of <15 were filtered out [22]. We then obtained functional or/and segregation analysis data on previously reported variants from both the human genome mutation database [23] and the ClinVar disease mutation database [24]. We additionally classified the remaining variants as one of the following: pathogenic, likely pathogenic, or variant of uncertain significance, according to the American College of Medical Genetics and Genomics (ACMG) consensus statement guidelines [25]. 

### 2.3. Statistical Analysis

Differences in the frequency of rare variants versus control variants archived in the Human Genetic Variation Database [26] and the Integrative Japanese Genome Variation Database [27] were assessed using Fisher’s exact test with *p* < 0.05 being statistically significant.

## 3. Results

### 3.1. Autopsy Cases

Fifteen cases showed myocyte disarray in the left ventricle (11 men and 4 women, aged 48–94 years, 74.1 ± 13.9 years). A summary of the clinical characteristics of all the cases is provided in Table 1. With regards to the cause of death, three cases died of illness (one died from aortic dissection, one from congestive heart failure, and one from advanced esophageal cancer), nine died by accident (three from drowning outdoors, one from drowning in a bathtub, one from hypothermia where the patient had a history of advanced dementia, two were killed in traffic accidents as pedestrians, one died in a house fire, and the remaining case died from a head injury after an accidental fall), and the other three cases were suicides. No cases were diagnosed with HCM while alive. Five cases had a history of hypertension, and four of these five cases were medicated. Arrhythmia was found in three cases; one of these cases had paroxysmal supraventricular tachycardia, and the other two cases had atrial fibrillation (Table 1).

### 3.2. Pathological Findings

**Figure 1:**
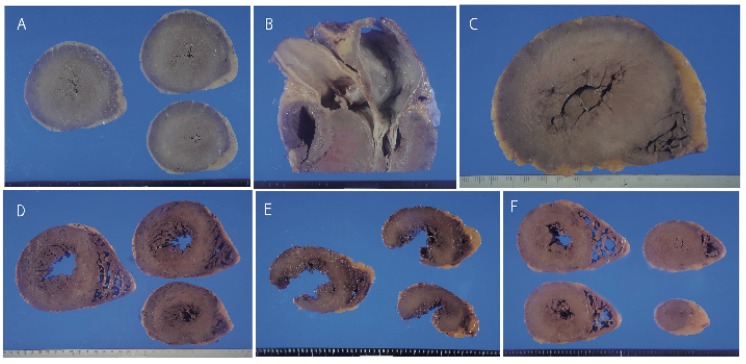
Gross appearance of the heart. **A**; Marked concentric left ventricular hypertrophy in Case 1. **B**; Asymmetric septal hypertrophy in Case 1. **C**; Marked concentric hypertrophy in Case 2. **D**; Moderate left ventricular hypertrophy in Cases 5 (**D**) and 6. E, F; Mild hypertrophy of the left ventricle in the hearts of Cases 7 (**E**) and 8 (**F**). The left lateral wall in Case 7 was ruptured by a traffic accident.

**Figure 2:**
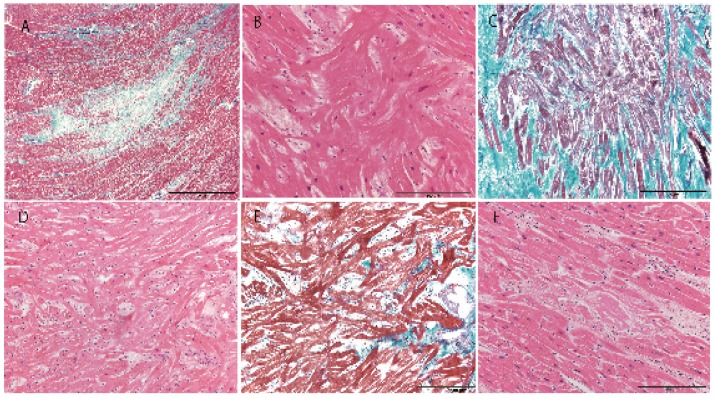
Histological appearance of the heart. **A**; Myocardial scar in Case 1 (Masson–trichrome). **B**; Myocyte disarray with hypertrophy of myocytes in Case 1 (hematoxylin–eosin). **C**; Myocyte disarray with severe interstitial fibrosis in Case 2 (Masson–trichrome). **D**; Myocyte disarray with mild myocyte hypertrophy in Case 7 (hematoxylin–eosin). **E**; Myocyte disarray with mild interstitial fibrosis in Case 8 (hematoxylin–eosin). **F**; Amyloidosis in Case 6 (hematoxylin–eosin). Scale bar = 2 mm (A), 200 µm (**B**–**E**).

A summary of all cases is described in Table 1. Three cases showed a heart weight >500 g. Left ventricular thickness was >1.5 cm in 10 cases and asymmetric septal hypertrophy was found in 2 cases (Figure 1). Myocyte disarray was found in the ventricular septum in 14 of 15 cases. Myocyte disarray was also found in the anterior wall in 10 cases, in the lateral wall in 5 cases, in the posterior wall in 2 cases, in the apical region in 2 cases, and in the right ventricle in 2 cases (Figure 2, Supplemental Figure S2). The extent of myocyte disarray in the whole specimen at the level of the papillary muscle was >10% in 6 cases, between 5% and 10% in 3 cases, and <5% in 6 cases. While interstitial fibrosis was found in all cases and the degree varied, the severity tended to increase with an increase in cardiac weight. One case showed old myocardial infarction with advanced coronary artery disease and another one case had transthyretin-positive mild senile amyloidosis. Three cases had narrowing of the coronary artery due to atherosclerosis, and 1 of the 3 cases showed healed plaque disruption and old myocardial infarction of the left ventricle. Fatal acute aortic dissection and advanced prostatic carcinoma with extensive lung metastasis and advanced esophageal cancer was found in one case each. Acute myocardial necrosis, myocarditis, and advanced valvular disease were not evident. Four cases showed advanced neurodegenerative diseases.

### 3.3. Genetics

In 7 (46.7%) of the 15 cases, no potential pathogenic mutation was identified. In 8 (72.7%) cases, a total of 8 rare variants in 5 different genes were identified; *MYBPC3* was the most frequently involved (4 variants in 4 cases, 2 variants in 1 case) and the remaining 4 genes (*CAV3*, *PRKAG2*, *MYH6*, *MYH7*, 4 cases, 4 variants) accounted for a lower frequency. All variants were missense. According to ACMG guidelines [25], 2 of the 8 rare variants were classified as pathogenic or likely pathogenic rare variants in 3 (20.0%) cases. These variants were MYBPC3_p.Arg470Gln and MYBPC3_p.Thr1046Met. The remaining 6 variants were classified as potentially pathogenic variants. Two rare heterozygous variants were found in 1 of the 15 (6.7%) cases (Table 1). We also found many rare missense variants of *TTN* (Supplemental Table S1). We evaluated these variants as benign in the present study because the actual pathogenic role of these *TTN* missense variants remains unknown. While Lopes et al. [28] found 219 *TTN* rare variants and 209 of them were novel missense variants, this cohort of individuals potentially had a sarcomeric gene mutation that likely caused HCM [28]. The details of detected rare variants are listed in Supplemental Table S2.

### 3.4. Phenotype–Genotype Correlation

In 6 cases with myocyte disarray of >10%, 5 (83.4%) had HCM-related rare variants. While all 3 cases with myocyte disarray of 5% and 10% had a rare variant, both cases with <5% of myocyte disarray did not have any such variants. The rate of cases with rare variants in those with myocyte disarray >5% was 86.7%. In 4 cases with *MYBPC3* variants, the cardiac weight, the amount and distribution of myocyte disarray, the degree of fibrosis, and thickness of the left ventricular wall were all varied.

## 4. Discussion

With few exceptions, many forensic autopsies in Japan are performed under the Criminal Code. These autopsies are typically performed when the cause of death is suspected to be unnatural or is possibly linked to a crime [17]. We speculate that many unusual deaths, as examined in the present study, have not been investigated in previous clinical and/or pathological studies on detecting HCM without antemortem diagnosis. However, our forensic autopsy case series may not accurately represent the general Japanese population, but might be close to such a population. The present study might be useful for establishing the correct prevalence of undiagnosed HCM in the Japanese general population, and may be useful for showing the clinicopathological appearance of such undiagnosed HCM.

When investigating an autopsy cohort targeting HCM, careful pathological investigation of a certain area of the ventricular wall may be essential for confirming the presence or absence of myocyte disarray. This is because myocyte disarray in individuals with HCM tends to be regional [2]. Previous investigations have shown myocyte disarray not only in the hearts of individuals with congenital heart disease, [29] but also in normal adult hearts [30]. However, extensive myocyte disarray is considered to be a highly sensitive and specific marker for diagnosing HCM because of mild myocyte disarray in normal hearts [30,31]. Some previous studies have suggested there is myocyte disarray of at least 5% in the heart of HCM [32]. Davies then proposed that myocardial disarray of 10% is required for diagnosis [3]. However, Rose also suggested that myocardial disarray of 5% is too low a value for accurate diagnosis of HCM and may lead to a false positive diagnosis in a small number of patients [33]. According to these studies, 5 of 10 cases with myocyte disarray of >10% can be pathologically diagnosed as HCM. 

Integration of genetic testing into autopsy is a recent advancement [34,35]. This technology represents major progress in the ability to rapidly analyze a large panel of HCM-related genes with the spread of NGS in clinical practice and autopsy [15,36]. Such advances require more specific expertise for appropriate interpretation of genetic results because a considerable number of sequence variants of unknown clinical or pathological significance was detected unexpectedly [37]. Previous genetic investigations showed that 35% to 60% of cases of clinically diagnosed HCM carried mutations in 1 of the sarcomere protein genes, [38,39,40] mainly *MYBPC3* and *MYH7* variants (70% of identified mutations) [15]. Furthermore, non-sacromeric genetic variants have been observed in approximately 25% of children with HCM [41]. While “background noise” of human genetic variation has increased with variants of uncertain significance by comprehensive genetic investigation using NGS, [42] the number of genes involved in HCM has increased with progress in genetic investigation [15,36]. Coincidence between phenotype and genotype may be important for exploring the pathogenicity for a rare variant with uncertain significance.

Eight HCM-related variants that were detected in the present study could be interpreted as pathogenic because of the association with a certain amount of myocyte disarray in the heart. Among the three variants found in cases 1, 8, and 9 with myocyte disarray between 5%-10%, *MYBPC3*_p.T1046M was identified as pathogenic according to the ACMG guidelines. Although the other two variants (found in cases 8 and 9) were of uncertain significance according to the ACMG guidelines, these variants were classed as likely pathogenic by either ClinVar or HGMD. However, 6 cases with myocyte disarray of <5% did not have any possible pathogenic variant. On the basis of previous pathological studies on myocardial disarray [3,29,30] and the results of the present study, we propose that coexistence of myocyte disarray of >5% and HCM-related rare variants may be diagnosed as HCM in postmortem investigations, as well as cases with myocyte disarray of 10%. If we applied this diagnostic index, 9 of 15 cases (Cases 1 to 9, 7 men and 2 women, aged 48-94 years, 75.0 ± 14.7 years) could be diagnosed with HCM, and the prevalence of HCM in the present autopsy cohort study was 0.65%. The relatively higher prevalence of HCM in the present case series than in previous clinical cohort studies (0.16–0.29%) [36] might be due to extraction of cases with myocyte disarray, despite lacking significant left ventricular hypertrophy in our autopsy cohort. Detection of HCM-related rare variants may enable HCM to be missed in postmortem examinations, especially when the clinical history and/or typical pathology of HCM pathology is not evident. Concomitant administration between pathological examinations and genetic investigations may increase the rate of accurate diagnosis of HCM at postmortem.

Clinical manifestations of HCM widely vary. Large cohorts of patients with HCM have clinically demonstrated a nearly normal life. The prevalence of death due to HCM may not be higher than expected, especially in older cases, [43] and only a minority of patients experience sudden cardiac death (SCD) [44]. Approximately, 5% to 10% of patients with HCM progress to end-stage disease with inherited systolic function and dilatation [15]. In the present study, 9 HCM cases did not include those with a younger onset or severe clinical signs possibly related to HCM. Only 1 case showed cardiac symptoms while alive, and possible HCM-related death was only found in 2 cases (Case 3 with congestive heart failure and Case 6 with sudden death in the bathtub). However, these 2 cases were older than 90 years, and each had additional disease, such as mild senile cardiac amyloidosis and advanced cancer as an additional contributing factor for their death. Therefore, these 9 cases may be late-onset or mildly progressive HCM. Retrospective analysis of pathological and genetic manifestations of our detected cases might show the clinicopathological and genetic profile of late-onset or modest HCM. 

Clinicopathological correlations in HCM have not been well investigated yet. The extent of myocardial hypertrophy and left ventricular outflow obstruction do not predict SCD [1,45]. Dimitrow and Dubiel showed through detail echocardiographic investigation that a significant morphological imbalance between hyperprophied ventricular septum with reversed curvature and narrowed left ventricle allowed identification of a subgroup of patients with HCM who have an increased risk of SCD [46]. While some studies have shown that myocardial scarring may be associated with an increased risk of ventricular arrhythmia [47,48], myocardial scarring is common in asymptomatic or mildly symptomatic patients with HCM [49]. The guidelines for HCM diagnosis proposed by the European Society of Cardiology use a sudden cardiac death score to predict the 5-year risk of sudden cardiac death; both myocardial thickness and the left ventricular outflow tract gradient are taken into consideration in the score [50]. Notably, there have been cases of SCD due to HCM without major pathology other than myocyte disarray [9]. Although our examined cases were limited, the pathological appearance of the present undiagnosed HCM cases, including heart weight, thickness of the left ventricle and degree of fibrosis, also varied. Additionally, 3 cases showed marked advanced myocardial hypertrophy with a cardiac weight >500 g and microscopic disorganization of the myocardium, similar to clinically diagnosed HCM cases as shown by previous investigations [2,20]. The clinicopathological diversity in the present study suggests the need for distinct pathological diagnostic criteria for HCM. 

Although the presence of diseases causing gene mutations is related to a worse prognosis, [51] the variants found in the present HCM cases should be evaluated as important, but modest, pathogenic variants. Information on the genotype–phenotype correlation of HCM, including clinical appearance and outcomes, is limited. An example of this situation is that although the *MYH7* mutation was initially related to a worse outcome than the *MYBPC3* mutation, [52] recent studies failed to show a significant difference in phenotype between these 2 groups [40,53]. Additionally, the *MYBPC3* mutation is associated with late onset, mild hypertrophy, a low incidence of SCD, and a relatively benign clinical course [53,54,55]. However, a recent investigation of Japanese patients showed that HCM cases with the *MYBPC3* mutation may have a high frequency of ventricular arrhythmia and syncope [56]. The diversity of clinicopathological manifestation of HCM suggests that the clinical course of each patient, including the prognosis, cannot usually be determined only by genetic analysis. The morphological, histological, and clinical phenotype of HCM is considered to be the consequence of complex interactions among a large number of determinants, ranging from causal genetic mutations to environmental factors [36]. The differences in pathological appearance, other than myocyte disarray between Cases 1 and 6 that commonly had *MYBPC3*_p.T1046M, strongly support this hypothesis. 

### Limitations

The number of HCM cases in the present study was limited. Therefore, we cannot determine whether cases with myocyte disarray of <5% can be completely ruled out from the diagnosis of HCM, especially when such cases have HCM-related pathogenic variants. Additionally, a causal role in HCM of *PRKAG2*, *CAV3*, and *MYH6,* which were detected in the present study, has not been well investigated because of the low prevalence of corresponding cases [36]. However, pathological changes in 3 cases showed that these variants are high and are possibly HCM-related pathogenic variants. Investigation for one case that a disease causing variant could not be found will be also needed. In the future, a study targeting the effect of overlapping common variants [57] or copy number variants, [55] which has been recently observed as a disease-causing factor, may also be required.

## 5. Conclusions

The resent study showed that cases with a certain amount of myocardial disarray (>5% in the heart) frequently had pathogenic HCM-related rare variants (8/9 cases, 88.9%). Therefore, these cases may be diagnosed as HCM, despite the presence or absence of clinical and/or pathological signs that are suggestive of HCM. The prevalence of undiagnosed HCM cases in the Japanese general population was 0.65% under our proposed postmortem diagnostic criteria. Many detected genetic variants in the present cases might have lower pathogenicity than the variants found in SCD or in other cases with severe clinical symptoms. Performing pathological investigations of a more extensive area in the heart and more advanced genetic investigation methods might enable a more definite diagnosis of HCM, and may clarify phenotype–genotype correlations in HCM more clearly.

## Figures and Tables

**Table 1:** Summary of clinical and autopsy findings.

Case	Age (y)	Sex	BMI (kg/m^2^)	Cause of Death	Clinical History	HW (g)	ASH	LV (cm)	Distribution of Myocyte Disarray						Area of Disarray (%)	Fib	Other Pathology	Variant	
									A	L	P	S	Ap	RV				Pathogenic/likely Pathogenic	Uncertain Significance
1	53	M	25.9	Head injury (suicide)	HT, PSVT (5 y)	726	+	2.5	+	-	+	+	+	+	8.5	3	CA (50% in the RCA and LAD)	*MYBPC3*_p.T1046M	-
2	71	F	27.7	Aortic dissection	Brain injury (25 y)	550	+	2.5	+	+	-	+	-	-	15.2	3	Aortic dissection	*MYBPC3*_p.R470Q	-
3	94	M	25.9	CHF	HT, dementia, prostatic cancer,	490	-	2.2	+	-	-	+	-	-	13.1	3	CA (60% in the LMT), Alzheimer’s disease	-	-
4	74	M	20.9	Drowning (accident)	af, gait disorder	481	-	2.0	-	-	-	+	-	+	14.2	2	CA (healed plaque rupture in the LAD), OMI, PSP	-	*MYH6*_p.D629N
5	72	M	20.6	Drowning (accident)	HT (20 y)	476	-	2.0	+	+	-	+	-	-	18.1	2	NS	-	*PRKAG2*_p.G75A
6	94	F	20.6	Drowning in the bathtub	Hyperuricemia	423	-	1.6	+	-	-	+	-	-	12.5	2	Cardiac amyloidosis(senile)	*MYBPC3*_p.T1046M	*MYBPC3*_p.R1138C
7	78	M	18.0	Multiple injuries (traffic accident)	NS	330	-	1.3	+	+	+	+	-	-	13.7	1	Prostatic cancer with pulmonary metastasis	-	*CAV3*_p.R148Q
8	48	M	20.1	Multiple injuries (traffic accident)	NS	320	-	1.2	+	+	-	-	-	-	7.0	1	NS	-	*MYBPC3*_p.E334K
9	69	M	25.2	Head injuries (accident)	NS	624	-	1.8	-	-	-	+	+	-	7.8	3	NS	-	*MYH7*_p.941H
10	75	M	18.1	Drowning (suicide)	Depression	450	-	1.8	+	-	-	+	-	-	2.4	1	PSP	-	-
11	86	F	NE	Burn (accident)	NS	300	-	1.3	-	-	-	+	-	-	1.8	1	NS	-	-
12	78	F	20.8	Drowning	HT, hypercholeste-rolaemia, angina pectoris, dementia	376	-	1.5	+	-	-	+	-	-	1.6	1	Chronic pancreatitis	-	-
13	83	M	27.1	Hypothermia	dementia	568	-	1.5	+	-	-	+	-	-	1.5	1	Esophageal cancer	-	-
14	81	M	26.0	CHF	Esophageal cancer	373	-	1.5	-	-	-	+	-	-	2.1	1	Lewy body disease	-	-

M, male; F, female; BMI, body mass index; HW, heart weight; ASH, asymmetric septal hypertrophy; LV, thickness of the left ventricle; A, left anterior wall; L, left lateral wall; P, left posterior wall; S, ventricular septum; RV, right ventricle; Fib, grade of fibrosis; HT, hypertension; PSVT, paroxysmal supraventricular tachycardia; CA, narrowing of the coronary artery; RCA, right coronary artery; LAD, left anterior descending artery; CHF, congestive heart failure; LMT, left main trunk; af, atrial fibrillation; OMI, old myocardial infarction; PSP, progressive supranuclear palsy; NS, not significant; NE, not examined.

## Supplementary Materials

The following are available online at https://www.mdpi.com/2077-0383/8/4/463/s1, Figure S1: Grading of fibrosis in the heart., Figure S2: Distribution of myocyte disarray; Table S1: List of the inherited cardiac disease genes analyzed in this study. Table S2; Pathogenic, likely pathogenic and possibly pathogenic rare variants in the study.
