# Supplementary material for: Clinicopathological and Genetic Profiles of Cases with Myocyte Disarray—Investigation for Establishing the Autopsy Diagnostic Criteria for Hypertrophic Cardiomyopathy
Source: J Clin Med. 2019 Apr 5;8(4):463. doi: 10.3390/jcm8040463 (PMC6518201; doi:10.3390/jcm8040463)
Supplement: Supplementary file 1 [file jcm-08-00463-s001.pdf]

Supplemental Figure 1. Grading of fibrosis in the heart. A; Grade 0, B; Grade 1, C; Grade 2, D; Grade 3. Scale bar = 500 m (A-C), 1cm (D).

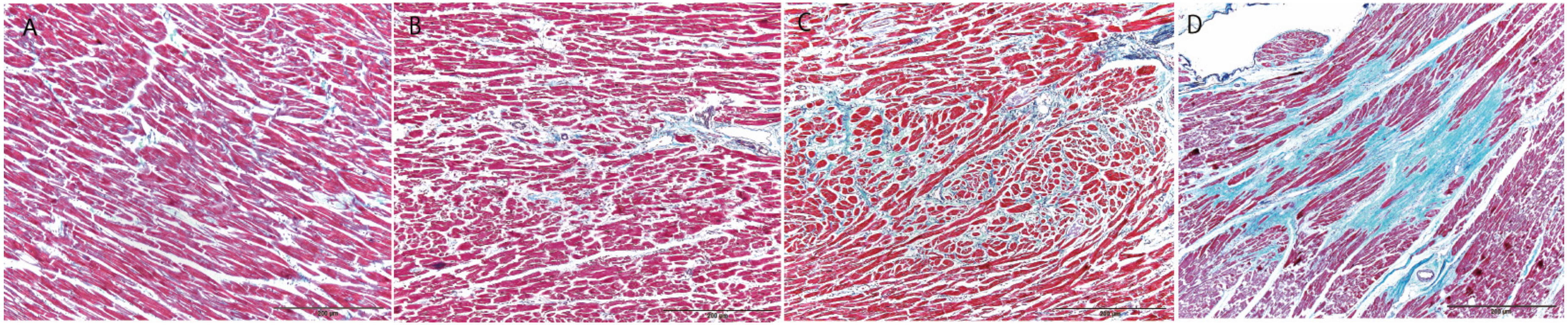

Supplemental Fig. 2. Distribution of myocyte disarray

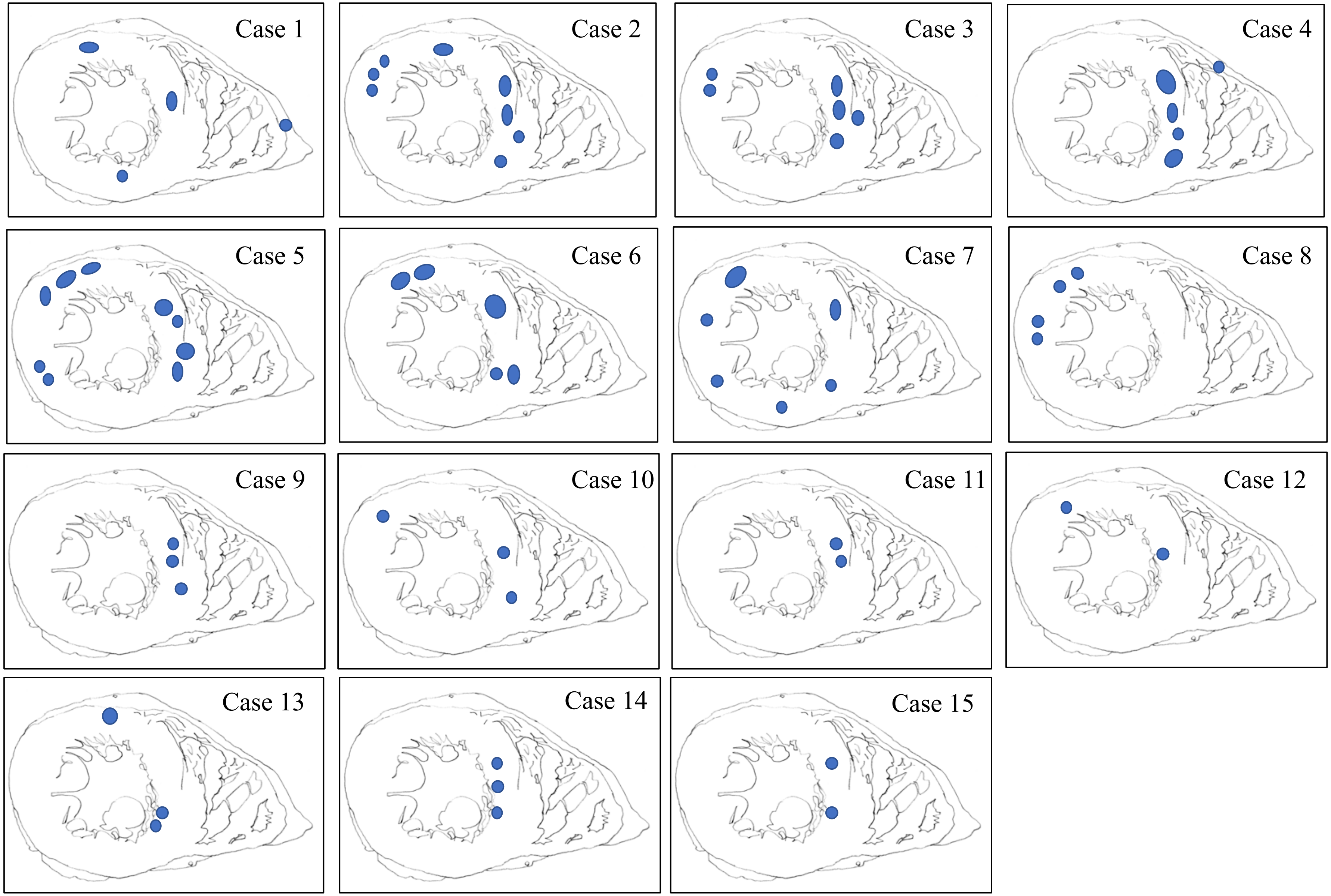

Supplementary Table 1. List of the inherited cardiac disease genes analyzed in this study.

Inherited cardiac disease-related (81 genes)

ABCC9, ACTC1, ACTN2, AKAP9, ANK2, ARFGEF2, BAG3, BMPR1A, CACNA1C, CACNB2, CALR3, CAPN3, CAV3, COL4A1, DES, DMD, DSC2, DSG2, DSP, ELN, EMD,, GAA, GATA4, GLA, GPD1L, HCN4, JUP, KCNE1, KCNE2, KCNE3, KCNH2, KCNJ2, KCNQ1, KRAS, LAMP2, LDB3, LMNA, MLYCD, MMACHC, MYBPC3, MYH11, MYH6, MYH7, MYL2, MYL3, MYLK, MYOZ2, NKX2-5, NNT, NRAS, NSD1, PKP2, PLEKHM2, PLN, PRKAG2, PTPN11, RAF1, RPS6KA3, RPS7, RYR2, SCN1B, SCN3B, SCN4B, SCN5A, SGCD, SLC25A4, SMAD3, SNTA1, SOS1, STARD3, TAZ, TBX5, TGFBR1, TGFBR2, TMEM43, TNNC1, TNNI3, TNNT2, TPM1, TTN, VCL

# locus	gene	transcript	protein	coding	dbSNP ID	gnomAD Frequency	HGVD	ToMMo	ACMG guidline Classification	ClinVar	HGMD	ID
Pathogenic and likely pathogenic variants												
chr11:47364429	MYBPC3	NM_000256.3	p.Arg470Gln	c.1409G>A	776734314	<Exomes>: ALL:0.00041% - AMR:0.0030%	-	-	Likely Pathogenic	Uncertain significance	CM116865 (DM); Cardiomyopathy, hypertrophic	2
chr11:47355161	MYBPC3	NM_000256.3	p.Thr1046Met	c.3137C>T	371061770	<Exomes>: ALL:0.0063% - EAS:0.070% - SAS:0.0032% - NFE:0.00090% - OTH:0.018%	1.03%	1.09%	Likely Pathogenic	Conflicting interpretations of pathogenicity	CM073210 (DM); Cardiomyopathy, hypertrophic	1, 6
**Possibly pathogenic variants for HCM**												
chr3:8787540	CAV3	NM_033337.2	p.Arg148Gln	c.443G>A	140575619	<Exomes+Genomes>: ALL:0.026% - AFR:0.11% - AMR:0.096% - EAS:0.021% - NFE:0.0048% - OTH:0.031%	0.08%	0.06%	Uncertain Significance	Conflicting interpretations of pathogenicity	-	7
chr7:151478480	PRKAG2	NM_016203.3	p.Gly75Ala	c.224G>C	199963585	<Exomes>: ALL:0.0046% - EAS:0.065%	-	0.18%	Uncertain Significance	Uncertain significance	-	5
chr11:47354443	MYBPC3	NM_000256.3	p.Arg1138Cys	c.3412C>T	377171707	<Exomes+Genomes>: ALL:0.0030% - AFR:0.018% - AMR:0.0030% - EAS:0.011% - NFE:0.00082%	0.08%	0.12%	Uncertain Significance	Uncertain significance	CM132315 (DM); Cardiomyopathy, hypertrophic	6
chr11:47367848	MYBPC3	NM_000256.3	p.Glu334Lys	c.1000G>A	573916965	<Exomes+Genomes>: ALL:0.024% - EAS:0.35% - NFE:0.00080%	0.58%	-	Uncertain Significance	Conflicting interpretations of pathogenicity	CM073211 (DM); Cardiomyopathy, hypertrophic	8
chr14:23867943	MYH6	NM_002471.3	p.Asp629Asn	c.1885G>A	180904654	<Exomes+Genomes>: ALL:0.0076% - EAS:0.080% - NFE:0.0024% - FIN:0.0078% - OTH:0.016%	0.25%	0.30%	Uncertain Significance	-	-	4
chr14:23893216	MYH7	NM_000257.3	p.Arg941His	c.2822G>A	765458590	<Exomes>: ALL:0.0016% - NFE:0.0018% - FIN:0.0090%	-	-	Uncertain Significance	Likely pathogenic	-	9
**Benign**												
chr1:236902765	ACTN2	NM_001103.3	p.Thr347Met	c.1040C>T	727504590	<Exomes+Genomes>: ALL:0.014% - AMR:0.023% - EAS:0.11% - SAS:0.0032% - NFE:0.0063% - OTH:0.031%	0.20%	0.30%	Likely Benign	Uncertain significance	-	13
chr2:179410980	TTN	NM_001256850.1	p.Ala30052Asp	c.90155C>A	2288326	<Exomes+Genomes>: ALL:0.018% - EAS:0.24% - SAS:0.0098%	0.99%	1.21%	Uncertain Significance	Conflicting interpretations of pathogenicity	-	4
chr2:179413687	TTN	NM_001256850.1	p.Gly29248Asp	c.87743G>A	727505280	<Exomes>: ALL:0.0012% - SAS:0.0032% - NFE:0.0018%	-	-	Uncertain Significance	Uncertain Significance	-	5
chr2:179425208	TTN	NM_001256850.1	p.Pro26910Thr	c.80728C>A	142478636	<Exomes+Genomes>: ALL:0.020% - EAS:0.25% - SAS:0.0065% - NFE:0.0040% - OTH:0.016%	0.83%	0.66%	Uncertain Significance	Likely benign	-	14
chr2:179427229	TTN	NM_001256850.1	p.Arg26236His	c.78707G>A	371345921	<Exomes+Genomes>: ALL:0.0047% - AFR:0.017% - AMR:0.0088% - SAS:0.0099% - NFE:0.0024%	-	0.01%	Uncertain Significance	Uncertain Significance	-	1
chr2:179427779	TTN	NM_001256850.1	p.Arg26053Cys	c.78157C>T	192360370	<Exomes+Genomes>: ALL:0.030% - AMR:0.0058% - EAS:0.39% - NFE:0.0040% - OTH:0.016%	1.36%	1.35%	Uncertain Significance	Conflicting interpretations of pathogenicity	-	11

chr2:179428277	TTN	NM_001256850.1	p.Arg25887Trp	c.77659C>T	749852593	<Exomes>: ALL:0.0033% - AMR:0.012% - SAS:0.0097% - NFE:0.00090%	-	-	Uncertain Significance	Uncertain Significance	-	6
chr2:179438056	TTN	NM_001256850.1	p.Arg22627His	c.67880G>A	140018785	<Exomes>: ALL:0.038% - AFR:0.014% - EAS:0.44% - NFE:0.0011%	1.16%	1.14%	Likely Benign	Conflicting interpretations of pathogenicity	-	6, 8
chr2:179439490	TTN	NM_001256850.1	p.Arg22149His	c.66446G>A	55677134	<Exomes+Genomes>: ALL:0.12% - AMR:0.012% - EAS:1.63% - SAS:0.020% - NFE:0.0040% - OTH:0.063%	3.80%	3.56%	Likely Benign	Benign/Likely benign	-	2, 4
chr2:179439952	TTN	NM_001256850.1	p.Arg21995His	c.65984G>A	56071233	<Exomes+Genomes>: ALL:0.13% - AFR:0.0042% - AMR:0.0029% - EAS:1.70% - SAS:0.065% - NFE:0.0080% - OTH:0.11%	3.47%	4.23%	Likely Benign	Conflicting interpretations of pathogenicity	-	3
chr2:179480434	TTN	NM_001256850.1	p.Arg14491Cys	c.43471C>T	2303830	<Exomes+Genomes>: ALL:0.016% - AFR:0.0083% - AMR:0.0029% - EAS:0.18% - NFE:0.00079% - FIN:0.012% - OTH:0.062%	1.90%	1.73%	Uncertain Significance	Conflicting interpretations of pathogenicity	-	1, 4, 9
chr2:179486408	TTN	NM_001256850.1	p.Ile13407Arg	c.40220T>G	727505261	-	-	-	Uncertain Significance	-	-	8
chr2:179486605	TTN	NM_001256850.1	p.Val13374Glu	c.40121T>A	786205390	-	0.08%	0.10%	Uncertain Significance	Uncertain Significance	-	8
chr2:179500729	TTN	NM_001256850.1	p.Ala12216Thr	c.36646G>A	144963490	<Exomes>: ALL:0.0037% - EAS:0.029% - NFE:0.0036%	0.08%	0.23%	Uncertain Significance	-	-	13
chr2:179547564	TTN	NM_001256850.1	p.Arg10668Pro	c.32003G>C	181395238	<Exomes+Genomes>: ALL:0.046% - EAS:0.66% - NFE:0.00079% - OTH:0.016%	1.37%	1.72%	Likely Benign	Conflicting interpretations of pathogenicity	-	11
chr2:179606622	TTN	NM_001256850.1	p.Glu3463Lys	c.10387G>A	727504586	<Exomes>: ALL:0.014% - AMR:0.021% - EAS:0.036% - SAS:0.021% - NFE:0.0094% - OTH:0.050%	0.29%	0.39%	Uncertain Significance	Conflicting interpretations of pathogenicity	-	2
chr2:179635985	TTN	NM_001256850.1	p.Thr2690Ile	c.8069C>T	374620001	<Exomes+Genomes>: ALL:0.057% - EAS:0.74% - SAS:0.049% - OTH:0.062%	0.29%	0.56%	Likely Benign	Uncertain significance	CM1811455; Cardiomyopathy, dilated	13
chr2:179641360	TTN	NM_001256850.1	p.Pro1744Leu	c.5231C>T	75686037	<Exomes+Genomes>: ALL:0.67% - AFR:0.021% - AMR:3.59% - ASJ:0.069% - EAS:2.70% - SAS:0.071% - NFE:0.030% - FIN:0.016% - OTH:0.48%	2.25%	1.99%	Likely Benign	Benign/Likely benign	-	1
chr10:75867074	VCL	NM_014000.2	p.Asp841His	c.2521G>C	150385900	<Exomes+Genomes>: ALL:0.11% - AFR:0.029% - EAS:1.53% - SAS:0.026% - NFE:0.00079% - OTH:0.093%	0.29%	0.30%	Likely Benign	Conflicting interpretations of pathogenicity	-	6
chr11:47355475	MYBPC3	NM_000256.3	p.Gln998Glu	c.2992C>G	11570112	<Exomes+Genomes>: ALL:0.64% - AFR:0.037% - AMR:3.94% - EAS:1.66% - SAS:0.049% - NFE:0.0066% - OTH:0.50%	0.83%	0.98%	Benign	Benign/Likely benign	CM043548 (DM) ; Cardiomyopathy, hypertrophic;	7

chr14:23863498	MYH6	NM_002471.3	p.Ala822Thr	c.2464G>A	138419275	<Exomes>: ALL:0.011% - AMR:0.0030% - EAS:0.14% - OTH:0.018%	0.54%	0.74%	Uncertain Significance	Likely benign	-	12

Supplemental Table 2 Pathogenic, likely pathogenic and possibly pathogenic rare variants in the study.
